# H3K27M Mutation Doesn’t Mean Worse Prognosis in Old Patients

**DOI:** 10.3389/fonc.2022.912166

**Published:** 2022-06-08

**Authors:** Xiao Mu Hu, Xiao yu Nie, Kai lun Xu, Yin Wang, Feng Tang, Zun guo Du, Ji Xiong

**Affiliations:** ^1^ Huashan Hospital, Fudan University, Shanghai, China; ^2^ Yueyang Hospital of Integrated Traditional Chinese and Western Medicine, Shanghai University of Traditional Chinese Medicine, Shanghai, China; ^3^ Xinhua Hospital, School of Medicine, Shanghai Jiao Tong University, Shanghai, China

**Keywords:** midline glioma, H3K27M, clinicopathological study, prognosis, age

## Abstract

**Objective:**

Diffuse midline glioma (DMG), H3K27 altered is a new entity that has become widely recognized. However, studies concerning DMG in adult patients remain rare. We did a retrospective study covering the largest amount of patients to date to analyze the clinicopathological characteristics of diffuse glioma in midline structures of the adult.

**Methods:**

We reviewed 108 cases of adult DMG, collected their clinical data, and pathological results including H3K27 mutation. Summarized their features and the connection with overall survival in different age groups.

**Results:**

Among 108 cases, 79 tumors were located at the thalamus. 38 patients had H3K27M mutation, whose average age was 35.7 years. The median overall survival of H3K27M-mutant gliomas and the 70 H3K27M wild-type gliomas were both 17 months. For young patients (age ≤ 35), The median survival time of the H3K27M-mutant was 18 months, while that of the H3K27M wild-type was 37 months. For older patients (age>35), the median survival time of the H3K27M-mutant was 16 months, while that of the H3K27M wild-type was 13 months. Other clinicopathological factors including sex, tumor location, the approach of surgery, histological grade, ATRX, and P53 were statistically irrelevant to prognosis.

**Conclusion:**

The DMG in adults mainly occurred in the thalamus. H3K27M mutations tend to happen more frequently in young adults, and this genetic alteration results in a worse outcome only in young patients (≤35). For old patients, age is the only independent prognostic factor. Patients who underwent different surgical operations including biopsy, subtotal resection, and total resection had similar prognoses.

## Introduction

The Diffuse Midline Glioma (DMG), H3K27 altered, is a new pathologic entity that has become widely recognized since it was first classified as a central nervous system neoplastic disorder by the WHO in 2016. As illustrated by its name, these gliomas are commonly located in midline structures of the brain, including the brainstem, thalamus, spinal cord, and cerebellum. These are defined as grade 4 gliomas and are correlated with worse prognoses (1). However, with more and more investigations being carried out on this tumor entity, several questions have been noticed by researchers. Firstly, due to this kind of glioma mainly involving children, cases in adult patients are seldom reported and their prognoses remain unclear. Whether the H3K27M mutation still indicates a poor prognosis is questionable. Secondly, carrying out successful neurosurgery to resect tumors in midline structures is a challenging task. As a result, the limitation on operations or genetic alterations that mainly contribute to the dismal outcome of DMG has been confused.

To figure out the prognosis of DMG in adult patients and explore the benefit that tumor resections offer patients, we have reviewed patients diagnosed with DMG in our hospital, collected their clinical and pathological data, and summarized its characteristics.

## Methods and Materials

### Study Cohort

In this retrospective study, we reviewed our pathology database and picked out cases of patients diagnosed with glioma in the midline structures of their brains - including the thalamus, brainstem, cerebellum, and spinal cord - from January 2013 to December 2019. All the patients in our cohort were adults (age>18), and had to satisfy the following standards: 1. no previous medical history of glioma; 2. no severe complications during the perioperative period; 3. possess a complete medical history and have an accessible survival outcome. The tumor locations were verified *via* a radiologist through preoperative MR images. Pathologic sections were reassessed by two experienced pathologists according to the 2021 WHO classification of central nervous system tumors. To confirm the diagnosis, the H3K27M status was tested or reconfirmed *via* immunostaining and PCR tests. All the tumor samples underwent genetic tests for IDH1/2. Other important markers including ATRX, P53, and Ki67 were all re-evaluated *via* immunohistochemistry.

By looking up the patients’ detailed operation records in our hospital’s medical history system and consulting the related neurosurgeons, we obtained the approaches of operations and range of resections. And the range was further confirmed from postoperative MR images by radiologists. We divided the patients into 3 subgroups according to the range of tumor resections: biopsy, subtotal resection, and total resection.

### Immunohistochemistry and Sanger Sequencing

All the tumor specimens were formalin fixed and paraffin embedded (FFPE). Immunohistochemistry was performed on 4um sections with the antibodies: H3K27M (ZSGB-BIO China, ZA-0321), H3K27me3 (ZSGB-BIO China, ZA-0327), IDH (ZSGB-BIO China, ZM-0447), ATRX (ZSGB-BIO China, ZA-0016), P53 (ZSGB-BIO China, ZM-0408), and Ki67 (Dako Denmark, GA626). For the PCR tests, DNA extractions were conducted with four 10mm sections from FFPE tissues. The enriched areas in tumor cells confirmed from HE sections were marked in advance. Tissues previously labeled for extraction were scraped off and placed on an Eppendorf tube for DNA isolation using the QIAamp DNA Mini Kit (Qiagen GmbH, Germany), the process for which was carried out according to the manufacturer’s protocol. A spectrophotometer (Biophotometer Eppendorf, Germany) was used for detecting the quality and concentration of DNA samples.

The sequencing reactions of Histone H3F3A and IDH1/2 were analyzed by direct sequencing of polymerase chain reaction–amplified products from tumor DNA using the primers designed according to literature (2, 3). Tests were performed using clean-up exonuclease ExoSAP-IT (Affymetrix, Santa Clara, CA) with a Big Dye Terminator Cycle Sequencing Kit and capillary electrophoresis on the automated sequencer ABI3730 (Applied Biosystems, Carlsbad, CA). The PCR products were then analyzed and purified. Exonic alterations of sense and nonsense sequences were detected using SeqScape v2.5 software (Applied Biosystems), before being compared with the National Center for Biotechnology Information reference sequences of H3F3A and IDH1/2.

### Statistical Analysis

We carried out statistical analysis using the STATA software (Stata Corp TEXAS V15.1). The Kaplan-Meier estimator and Log-Rank test were conducted to form a comparison of patients’ survival rates in different subgroups. We also performed univariate and multivariate COX models to evaluate the significance of related factors. Spearman test was done to verify the correlation between age and H3K27M mutation. The χ^2^ test, Fisher’s exact t-test, and Welch’s t-test were done to compare differences in clinicopathological factors in subgroups divided by H3K27M mutation. The OS was defined and counted from the date of surgery to the date of death from any causes. This is viewed as statistically significant when p<0.05.

## Results

### Epidemiology of Patients

**Figure 1:**
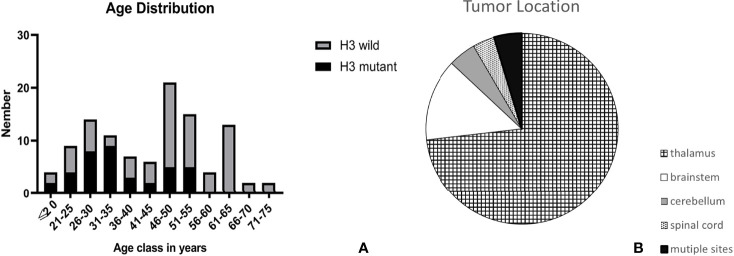
The age **(A)** and location **(B)** distribution of tumor in our research.

**Figure 2:**
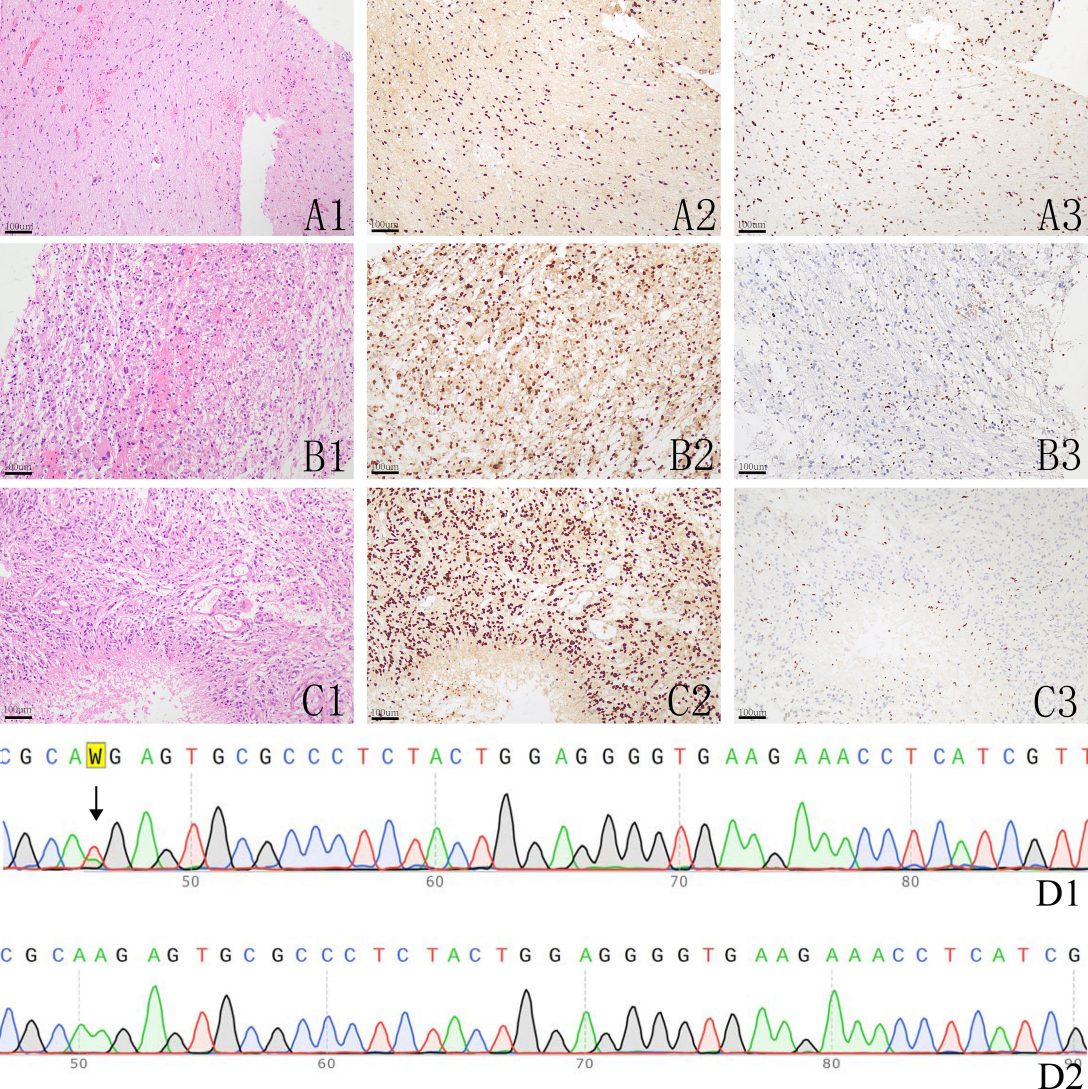
The histological image and immunohistochemistry results of H3K27M and H3K27me3, **A1-A3**: a histologically grade 2 glioma showing H3K27M expression **(A2)** and downregulation of H3K27me3 **(A3)**; **B1-B3**: a histologically grade 3 glioma showing H3K27M expression **(B2)** and slightly downregulation of H3K27me3 **(B3)**; **C1-C3**: a histologically grade 4 glioma showing H3K27M expression **(C2)** and loss the expression of H3K27me3 **(C3)**; **D1-D2**: Sanger sequencing of H3F3A in one H3K27M mutant glioma showing A > T mutation **(D1)** and a H3K27M wild glioma **(D2)**.

We identified 108 patients with DMG. They were all adults (>18 years old) ranging from 18 to 74 years old, with an average age of 43.5. 61 were males and 47 were females. Their pathological and clinical characteristics are summarized in **Table 1**. 79 tumors were located in the thalamus, 15 tumors in the brainstem, 5 in the cerebellum, and 4 in the spinal cord. The remaining 5 tumors involved multiple sites mentioned above (**Figure 1**). Histologically, 19 corresponded to WHO grade 2, 23 were grade 3, and the remaining 66 cases were grade 4 (**Figures 2A1, B1, C1**).

**Table 1:** Clinical and pathological characteristics of the patients with gliomas in midline location.

		H3K27M-Wild(N=70)	H3K27M-mutant(N=38)	P-value
**Epidemiology**				
Sex				
M	36 (33.3)		25 (23.1)	*P*=0.151
F	34 (31.5)		13 (12.1)	
Age (Y)				
≤20	2 (1.9)		2 (1.9)	*P*=0.005
21-25	5 (4.6)		4 (3.7)
25-30	6 (5.6)		8 (7.4)
31-35	2 (1.9)		9 (8.3)
36-40	4 (3.7)		3 (2.8)
41-45	4 (3.7)		2 (1.9)
46-50	16 (14.8)		5 (4.6)
51-55	10 (9.3)		5 (4.6)
56-60	4 (3.7)		0
61-65	13 (12.0)		0
66-70	2 (1.9)		0
71-75	2 (1.9)		0
Location				
Brainstem	7 (6.5)		8 (7.4)	*P*=0.272
Thalamus	52 (48.1)		27 (25.0)
Cerebellum	3 (2.8)		2 (1.9)
Spinal cord	3 (2.8)		1 (0.9)
Multiple sites	5 (4.6)		0
**Pathology**				
WHO Histology Grading				
2	16 (14.8)		3 (2.8)	*P*=0.051
3+4	54 (77.1)		35 (32.4)
IDH Mutation				
Yes	3 (2.8)		0	*P*=0.293
No	67 (62.0)		38 (35.2)
ATRX Mutation				
Yes	17 (15.7)		16 (14.8)	*P*=0.055
No	53 (49.1)		22 (20.4)	*P*=0.055
P53 Overexpression				
Yes	32 (29.6)		21 (19.4)	*P*=0.871
No	37 (34.3)		17 (15.7)
H3K27Me3 Downregulation				
Yes	0		38 (35.2)	*P*=0.000
No	70 (64.8)		0
**Surgical resection**				
Biopsy	29 (26.9)		13 (12.0)	*P*=0.023
Subtotal resection	27 (25.0)		22 (20.4)
Total resection	14 (13.0)		3 (2.8)
**Median Overall Survival**				
Young (M)	37		18	*P*=0.812
Old (M)	16		13

Y, years; M, months; The figures in the brackets are percentage of all patients.

### Immunohistochemistry and Genetic Findings

According to our IHC results, 38 patients were positive for H3K27M, and these tumors all demonstrated down regulation of H3K27me3. Out of the tumors that were negative for H3K27M, 70 samples were all diffusely positive for H3K27me3 (**Figure 2**). All tumor samples underwent PCR tests for H3.3 K27M, and all H3K27M immune-positive gliomas were genetically verified to be H3K27M mutants (**Figure 2D**). Those who were H3K27M negative were confirmed to be H3K27M wild-type. The mutation rate of H3K27M in the thalamus, brainstem, cerebellum, spinal cord and multiple locations was 34.2%, 53.3%, 20%, 25.0%, and 0% respectively. There were a total of 23 (23/38 50.5%) H3K27M mutant cases in the young adult group and 15 (15/70 21.4%) in the old group. The mutation rate of H3K27M was also included in **Table 1**. Spearman tests demonstrated that there existed a weak correlation between age and H3K27M mutation (*p*=0.394). G34R/V mutation was not detected in our cohort. We also conducted routine IDH1/2 tests for all tumor specimens. Only 3 gliomas were mutated at R132H of IDH-1, and the rest were all IDH wild-type. The 3 IDH mutant gliomas were in the cerebellum (n=1) and brainstem (n=2), and were all H3K27M wild-type. In our cohort, 58 gliomas (53.7%) showed overexpression of P53, and 33 (30.6%) gliomas had lost the expression of ATRX. Loss of ATRX was observed in 16 H3K27M mutant gliomas and 17 H3K27M wildtype gliomas.

### Treatment and Overall Survival

Out of all the patients, 17 received a total tumor resection and 49 patients underwent a subtotal tumor resection; 42 patients received a stereotactic biopsy instead of a tumor resection. All patients received chemotherapy with temozolomide accompanied by radiotherapy.

**Figure 3:**
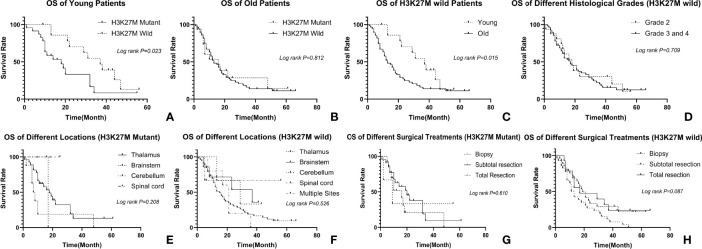
Relationship between clinicopathological factors and OS: **(A)** relationship between H3K27M and OS in young patients, the H3K27M mutant had a worse prognosis; **(B)** relationship between H3K27M and OS in old patients, there is no difference in prognosis of two different subgroups; **(C)** prognosis of different age groups in H3K27M wild patients, the young has a better prognosis. **(D)** relationship between histological grade and OS in H3K27M wild patients. There is no difference in different histological grades; **(E, F)** relationship between tumor location and OS in patients, tumors in different location have similar prognosis regardless of H3K27M mutation; **(G, H)** relationship between operation and OS in patients, patients underwent biopsy, subtotal resection or total resection have similar prognosis regardless of H3K27M mutation.

The mean follow-up time was 19.4 months (range from 7 to 66 months). Our patients’ OS ranged from 2 months to 56 months with a median survival time of 17 months. The 1-year and 3-year survival rates were 55.7% and 14.8% respectively. The median survival time of the young adults was 22 months, while that of the older patients was 13 months (*p*=0.244). For 38 H3K27M mutant gliomas, the median OS was 17 months, while that of 70 H3K27M wild-type gliomas was also 17 months (*p=0.986*). The median OS of 19 histologically low-grade gliomas (grade 2) was 18 months and that of 89 high-grade gliomas (grade 3 and 4) was 17 months (*p=0.353*). It can therefore be seen that there is no statistical difference between the subgroups divided by H3K27M mutation or histological grade, the same results can also be observed when tumors were divided by locations (**Figures 3D–F**). When dividing the data according to approaches of operations they received, the median OS of patients who received biopsies, subtotal resections, and total resections were 17, 14, and 17 months respectively. There is no statistical difference between the three groups (*p=0.303*) (**Figures 3G, H**).

We then further explored the prognosis in different age groups. We tried different ages from 25 to 70 by every gap of 5 years to explore an appropriate cutoff value to define age subgroups. We finally found that 35 was a statistically significant cutoff age. As a result, we divided our cohorts into two subgroups depending on whether their age was >35, which formed the old group, or ≤35, which formed the young adult group. Out of all the patients, 38 were classified as young adults, and 70 were put in the old group. Although having a similar outcome in the long run, the young have a better prognosis than the old for those H3K27 wild gliomas (**Figure 3C**). In younger patients, the clinicopathological factors mentioned above, including tumor location (*p=0.336*), sex (*p=0.694*), ATRX (*p=0.599*), P53 (*p=0.523*), histological grades (*p=0.348*), and operations (*p=0.062*) had no statistical relevance with prognoses. The median survival time of the H3K27M-mutant was 18 months, while that of the H3K27M wild-type was 37 months (*p=0.023*) (**Figure 3A**). For older patients, H3K27M was not a significant factor that affected patients’ outcomes since the median survival time of the H3K27M-mutant was 16 months, while that of the H3K27M wild-type was 13 months (*p=0.812*) (**Figure 3B**).

## Discussion

The “DMG, H3K27M mutant” is a newly defined entity in the 2016 revision of the WHO classification of central nervous system tumors. In the 2021 WHO classification, it was renamed as “DMG, H3K27 altered” and classified into pediatric type high grade glioma (4). It is a high grade glioma that mainly occurs in children, and the mutation in H3 often results in a dismal outcome, with a median survival of around 1 year from the time of diagnosis in children (5). However, the characteristics of this tumor in adults remain unclear. We carried out a retrospective analysis on 108 adult patients which focused on their clinical and pathological characteristics and overall survival. To date, this is the largest cohort concerning DMG in adults in available literature

Unlike pediatric patients whose midline gliomas are mostly located at the brainstem and pons (6), our results revealed that the most common sites of DMG in adults are the thalamus as the finding of the study by Huy Gia et al. (7). For H3K27M wild-type gliomas, histologically low grade cases comprised more than H3K27M mutated gliomas. The average age of H3K27M-mutated glioma patients was younger than the H3K27M wild-type patients. There was no H3K27M mutation detected in patients older than 55. According to our analysis, there is a weak correlation between H3K27M mutations and the age of patients. All these factors prove that the H3K27M mutation tends to happen in younger patients among all age ranges. The older a patient is, the lower the chance they will develop a H3K27M mutation. Another mutation of H3F3A, H3G34R/V, was not founded in our research, partly because the H3G34 mutation are mostly founded in diffuse hemispheric gliomas (8). Genetic tests concerning HIST1H3B/C were not included in this study. However, we believe that this had little effect on our result since H3K27me3, the trimethylated lysine 27 of histone could be downregulated by mutation of K27M in both H3.3 and H3.1 (9), were evaluated in all tumor samples and the rate of H3.1 K27M mutation in adult patients were lower than that of children (10).

Compared with the 6% rate of adult GBM (11)and the 14-29% rate of pediatric high grade glioma (12), the observed rate of ATRX mutation in our study was 42.1% and 24.3% in the H3K27M mutated gliomas and the H3K27M wild-type gliomas, respectively. The ratio was higher than gliomas in non-midline structures, corresponding to the fact that H3K27M mutated gliomas seem to predominantly result from lengthening of telomeres through ATRX mutation (13). Consistent with former reports, P53 overexpression was easily observed in our research, especially in the H3K27M mutated groups (14).

The H3K27M mutation is a genetic alteration reportedly linked with dismal outcomes in pediatric diffuse intrinsic pontine gliomas (DIPG) (15). The same phenomenon was also found in some studies concerning H3K27M mutated DMG in adults (16, 17). From 30 cases combined with the 171 cases reported in literature, Toshiyuki et al. found that the status of H3K27M is not related to the prognosis in patients who are older than 40 (18). However, another piece of research reported that H3K27M does not correlate with the prognoses of adults (19). To date, our study covers the largest cohorts and found that H3K27M mutant gliomas tend to have poorer prognoses in patients younger than 35. For patients older than 35, the H3K27M mutation does not affect the patients’ outcomes. The contradictory results found in the literature are possibly due to some research not subdividing patients according to age. Our cohort’s cutoff value for age was 35 instead of 40 used in the literature mentioned above (18). More cases are needed to discover the potential link between H3K27M and patients’ ages.

It is challenging for neurosurgeons to conduct operations on midline structures. The development of technologies - including the integration of functional mapping data on the neuro navigation cortical and subcortical electrical stimulation and awake anesthesia - has made surgical intervention more widely accepted as an important part of DMG treatment (20, 21).

It is widely accepted that extensive resections of high grade gliomas are beneficial for patients, with more extensive resections providing greater advantages (22). For adult DMG, it was reported that patients could benefit from tumor resections (23, 24). Our work found that patients who underwent different operations had a similar outcome, regardless of whether H3K27M had mutated or not. Our research was limited by the fact that we did not include a quantitative analysis of the extent of resections, and we didn’t take postoperative morbidity along with life quality into consideration. However, this tells us that if it is not necessary, neurosurgeons don’t need to risk a total tumor resection. Biopsies are recommended so that the genetic phenotype can be accurately decided at a low cost of living quality.

With regards to pediatric DMG, the WHO histological grade is of no prognostic significance (25). From our observations of adult patients, the tumor grade does not influence on the prognosis either. According to the 2021 WHO classification, all of our H3K27M wild-type gliomas that used to be diagnosed as “astrocytoma, grade II” may be considered to be diagnosed as glioblastoma now since they are all adult-type glioma and IDH wild-type. Due to the limitation of tumor samples from biopsy, we didn’t perform tests concerning some relatively rare genetic alterations whose significance is unclear in adult patients. Clinical significance of other forms of H3K27 mutation, EGFR amplification or mutation, overexpression of EZHIP, and TERT mutation are needed to be explored in future studies on DMG in adults. Our follow-up shows that our cohorts share a similar prognosis with glioblastoma in other sites (26).

It is interesting of the H3K27M mutation’s low frequency and meaninglessness in old patients. Further studies with complete genetic records are needed to describe the characteristics of this kind of glioma and potentially obtain a more comprehensive understanding of the gene, H3K27.

## Conclusions

In conclusion, this study covers the largest single-center series of gliomas in the midline structure of adult patients. It found that the DMG in adults mainly occurred in the thalamus. H3K27M mutations tend to happen more frequently in young adults (age<35), and this genetic alteration only results in a worse outcome in young patients. For old patients, DMG has a similar outcome as glioblastomas in cerebral hemisphere, and age is an independent prognostic factor, while H3K27M mutation has no prognostic value.

## Data Availability Statement

The raw data supporting the conclusions of this article will be made available by the authors, without undue reservation.

## Author Contributions

XH contribute to investigation and writing the original draft; XN contributed to investigation and data collection; KX mainly contributed to data analysis and design experiments; YW modified the manuscript; FT provided the method; ZD contributed to study design; JX designed the project and revised the manuscript. All authors contributed to the article and approved the submitted version.

## Funding

ZD was funded by Natural Science Foundation of China (Number: 82072692).

## Conflict of Interest

The authors declare that the research was conducted in the absence of any commercial or financial relationships that could be construed as a potential conflict of interest.

## Publisher’s Note

All claims expressed in this article are solely those of the authors and do not necessarily represent those of their affiliated organizations, or those of the publisher, the editors and the reviewers. Any product that may be evaluated in this article, or claim that may be made by its manufacturer, is not guaranteed or endorsed by the publisher.

## References

[1] Louis DN Perry A Reifenberger G von Deimling A Figarella-Branger D Cavenee WK. The 2016 World Health Organization Classification of Tumors of the Central Nervous System: A Summary. Acta Neuropathol (2016) 131:803–20. doi: 10.1007/s00401-016-1545-1 27157931

[2] Huang TY Piunti A Lulla RR Qi J Horbinski CM Tomita T. Detection of Histone H3 Mutations in Cerebrospinal Fluid-Derived Tumor DNA From Children With Diffuse Midline Glioma. Acta Neuropathol Commun (2017) 5:28. doi: 10.1186/s40478-017-0436-6 28416018 PMC5392913

[3] Perizzolo M Winkfein B Hui S Krulicki W Chan JA Demetrick DJ. IDH Mutation Detection in Formalin-Fixed Paraffin-Embedded Gliomas Using Multiplex PCR and Single-Base Extension. Brain Pathol (2012) 22:619–24. doi: 10.1111/j.1750-3639.2012.00579.x PMC8057634 22360629

[4] Louis DN Perry A Wesseling P Brat DJ Cree IA Figarella-Branger D. The 2021 WHO Classification of Tumors of the Central Nervous System: A Summary. Neuro Oncol (2021) 23:1231–51. doi: 10.1093/neuonc/noab106 PMC8328013 34185076

[5] Lowe BR Maxham LA Hamey JJ Wilkins MR Partridge JF. Histone H3 Mutations: An Updated View of Their Role in Chromatin Deregulation and Cancer. Cancers (Basel) (2019) 11(5):660. doi: 10.3390/cancers11050660 31086012 PMC6562757

[6] Solomon DA Wood MD Tihan T Bollen AW Gupta N Phillips JJ. Diffuse Midline Gliomas With Histone H3-K27M Mutation: A Series of 47 Cases Assessing the Spectrum of Morphologic Variation and Associated Genetic Alterations. Brain Pathol (2016) 26:569–80. doi: 10.1111/bpa.12336 PMC6055926 26517431

[7] Vuong HG Le HT Jea A McNall-Knapp R Dunn IF. Risk Stratification of H3 K27M–mutant Diffuse Midline Gliomas Based on Anatomical Locations: An Integrated Systematic Review of Individual Participant Data. J Neurosurg: Pediatr (2022) 29:1–8. doi: 10.3171/2022.3.PEDS2250 35535848 PMC10193490

[8] Wang L Shao L Li H Yao K Duan Z Zhi C. Histone H3.3 G34-Mutant Diffuse Gliomas in Adults. Am J Surg Pathol (2022) 46:249–57. doi: 10.1097/PAS.0000000000001781 34352809

[9] Lewis PW Muller MM Koletsky MS Cordero F Lin S Banaszynski LA. Inhibition of PRC2 Activity by a Gain-of-Function H3 Mutation Found in Pediatric Glioblastoma. Science (2013) 340:857–61. doi: 10.1126/science.1232245 PMC3951439 23539183

[10] Zheng L Gong J Yu T Zou Y Zhang M Nie L. Diffuse Midline Gliomas With Histone H3 K27M Mutation in Adults and Children: A Retrospective Series of 164 Cases. Am J Surg Pathol (2022) 46:863–71. doi: 10.1097/PAS.0000000000001897 PMC9093723 35416795

[11] Brennan CW Verhaak RG McKenna A Campos B Noushmehr H Salama SR. The Somatic Genomic Landscape of Glioblastoma. Cell (2013) 155:462–77. doi: 10.1016/j.cell.2013.09.034 PMC3910500 24120142

[12] Schwartzentruber J Korshunov A Liu XY Jones DT Pfaff E Jacob K. Driver Mutations in Histone H3.3 and Chromatin Remodelling Genes in Paediatric Glioblastoma. Nature (2012) 482:226–31. doi: 10.1038/nature10833 22286061

[13] Sturm D Bender S Jones DT Lichter P Grill J Becher O. Paediatric and Adult Glioblastoma: Multiform (Epi)Genomic Culprits Emerge. Nat Rev Cancer (2014) 14:92–107. doi: 10.1038/nrc3655 24457416 PMC4003223

[14] Aihara K Mukasa A Gotoh K Saito K Nagae G Tsuji S. H3F3A K27M Mutations in Thalamic Gliomas From Young Adult Patients. Neuro Oncol (2014) 16:140–6. doi: 10.1093/neuonc/not144 PMC3870821 24285547

[15] Castel D Philippe C Calmon R Le Dret L Truffaux N Boddaert N. Histone H3F3A and HIST1H3B K27M Mutations Define Two Subgroups of Diffuse Intrinsic Pontine Gliomas With Different Prognosis and Phenotypes. Acta Neuropathol (2015) 130:815–27. doi: 10.1007/s00401-015-1478-0 PMC4654747 26399631

[16] Meyronet D Esteban-Mader M Bonnet C Joly MO Uro-Coste E Amiel-Benouaich A. Characteristics of H3 K27M-Mutant Gliomas in Adults. Neuro Oncol (2017) 19:1127–34. doi: 10.1093/neuonc/now274 PMC5570304 28201752

[17] Liu Y Zhang Y Hua W Li Z Wu B Liu W. Clinical and Molecular Characteristics of Thalamic Gliomas: Retrospective Report of 26 Cases. World Neurosurg (2019) 126:e1169–e82. doi: 10.1016/j.wneu.2019.03.061 30885860

[18] Enomoto T Aoki M Hamasaki M Abe H Nonaka M Inoue T. Midline Glioma in Adults: Clinicopathological, Genetic, and Epigenetic Analysis. Neurol Med Chir (Tokyo) (2020) 60:136–46. doi: 10.2176/nmc.oa.2019-0168 PMC7073699 31902873

[19] Ebrahimi A Skardelly M Schuhmann MU Ebinger M Reuss D Neumann M. High Frequency of H3 K27M Mutations in Adult Midline Gliomas. J Cancer Res Clin Oncol (2019) 145:839–50. doi: 10.1007/s00432-018-02836-5 PMC11810378 30610375

[20] Alemo S Sayadipour A. Role of Intraoperative Neurophysiologic Monitoring in Lumbosacral Spine Fusion and Instrumentation: A Retrospective Study. World Neurosurg (2010) 73:72–6; discussion e7. doi: 10.1016/j.surneu.2009.04.024 20452872

[21] De Witt Hamer PC Robles SG Zwinderman AH Duffau H Berger MS. Impact of Intraoperative Stimulation Brain Mapping on Glioma Surgery Outcome: A Meta-Analysis. J Clin Oncol (2012) 30:2559–65. doi: 10.1200/JCO.2011.38.4818 22529254

[22] Tang S Liao J Long Y. Comparative Assessment of the Efficacy of Gross Total Versus Subtotal Total Resection in Patients With Glioma: A Meta-Analysis. Int J Surg (2019) 63:90–7. doi: 10.1016/j.ijsu.2019.02.004 30742934

[23] Wu B Tang C Wang Y Li Z Hu S Hua W. High-Grade Thalamic Gliomas: Microsurgical Treatment and Prognosis Analysis. J Clin Neurosci (2018) 49:56–61. doi: 10.1016/j.jocn.2017.12.008 29248381

[24] Dey M Lin Y Melkonian S Lam S. Prognostic Factors and Survival in Primary Adult High Grade Brainstem Astrocytoma: A Population Based Study From 1973-2008. J Clin Neurosci (2014) 21:1298–303. doi: 10.1016/j.jocn.2013.12.011 24674698

[25] Cohen KJ Jabado N Grill J. Diffuse Intrinsic Pontine Gliomas-Current Management and New Biologic Insights. Is There a Glimmer of Hope? Neuro Oncol (2017) 19:1025–34. doi: 10.1093/neuonc/nox021 PMC5570259 28371920

[26] Ostrom QT Cioffi G Gittleman H Patil N Waite K Kruchko C. CBTRUS Statistical Report: Primary Brain and Other Central Nervous System Tumors Diagnosed in the United States in 2012-2016. Neuro Oncol (2019) 21:v1–v100. doi: 10.1093/neuonc/noz150 31675094 PMC6823730

