# Corrigendum: H3K27M mutation doesn’t mean worse prognosis in old patients

**DOI:** 10.3389/fonc.2022.1061604

**Published:** 2023-01-12

**Authors:** Xiao Mu Hu, Xiao yu Nie, Kai lun Xu, Yin Wang, Feng Tang, Zun guo Du, Ji Xiong

**Affiliations:** ^1^ Huashan Hospital, Fudan University, Shanghai, China; ^2^ Yueyang Hospital of Integrated Traditional Chinese and Western Medicine, Shanghai University of Traditional Chinese Medicine, Shanghai, China; ^3^ Xinhua Hospital, School of Medicine, Shanghai Jiao Tong University, Shanghai, China

**Keywords:** midline glioma, H3K27M, clinicopathological study, prognosis, age

In the published article, there was an error. There was a data error in the abstract.

A correction has been made to the Abstract, *Results*, Paragraph 1. This sentence previously stated:

“The median overall survival of H3K27M-mutant gliomas and the 80 H3K27M wild-type gliomas were both 12 months”.

The corrected sentence appears below:

“The median overall survival of H3K27M-mutant gliomas and the 70 H3K27M wild-type gliomas were both 17 months”.

The authors apologize for this error and state that this does not change the scientific conclusions of the article in any way. The original article has been updated.

